# Application of multiplex PCR-based nanopore sequencing for rapid detection and surveillance of antimicrobial-resistant *Mycoplasma bovis* in Japanese dairy cattle

**DOI:** 10.1128/spectrum.01242-26

**Published:** 2026-06-22

**Authors:** Masaru Usui, Masashi Ebisawa, Shingo Okamura, Azul Noda, Akira Fukuda, Chie Nakajima, Yasuhiko Suzuki

**Affiliations:** 1 Laboratory of Food Microbiology and Food Safety, Department of Health and Environmental Sciences, School of Veterinary Medicine, Rakuno Gakuen University 13022, Hokkaido, Japan; 2 NDTS Co., Ltd., Sapporo-shi, Hokkaido, Japan; 3 Dairy Technology Research Institute, National Federation of Dairy Co-operative Association 13022, Yabukimachi, Fukushima, Japan; 4 Division of Bioresources, Hokkaido University International Institute for Zoonosis Control 12810, Sapporo, Hokkaido, Japan; 5 Division of Vaccinology for Clinical Development, Hokkaido University Institute for Vaccine Research and Development 12810, Sapporo, Hokkaido, Japan; 6 Division of Research Support, Hokkaido University Institute for Vaccine Research and Development 12810, Sapporo, Hokkaido, Japan; Taichung Veterans General Hospital, Taichung, Taiwan

**Keywords:** antimicrobial resistance, bovine mastitis, multiplex PCR, *Mycoplasma bovis*, nanopore sequencing

## Abstract

*Mycoplasma bovis* causes mastitis and respiratory disease, leading to economic losses in the dairy industry. Conventional culture and antimicrobial susceptibility testing require approximately 2 weeks, promoting empirical antimicrobial use and emergence of resistant strains, highlighting the need for rapid detection methods. Resistance-associated mutations have been detected in *rrl* and *rrs* (for macrolides, lincosamides, tetracyclines, and phenicols) and *gyrA* and *parC* (for fluoroquinolones), providing targets for a rapid prediction. We developed a multiplex PCR-based nanopore sequencing method capable of simultaneously analyzing up to 24 samples per run to detect mutations in *rrl*, *rrs*, *gyrA*, and *parC*, and infer the antimicrobial susceptibility of *M. bovis* within 6 h. Using 329 isolates from cattle collected in Japan (2013–2016 and 2023–2025), we linked genotypes to the minimum inhibitory concentrations of seven antimicrobials and predicted the susceptibility of all isolates. A clear association was observed between specific mutations and susceptibility to multiple antimicrobials, suggesting that susceptibility can be predicted based on target gene sequence. Subsequently, the temporal trends in the prevalence of resistance-associated mutations were examined annually. High-level macrolide- and tetracycline-associated mutations (*rrl* A2059/A2060, *rrs* G1058) and dual *gyrA-parC* mutations increased markedly in recent isolates, with frequencies rising from 1.8% to 43.3%, 0.9% to 41.5%, and 59.8% to 79.8%, respectively, during 2013–2016 and 2023–2025. This nanopore-based platform enables rapid genetics-based antimicrobial guidance and simultaneous analysis of up to 24 samples on a low-cost flow cell, providing a practical tool for clinical decision-making and surveillance of emerging multidrug-resistant *M. bovis* in dairy cattle.

**IMPORTANCE:**

*Mycoplasma bovis* is a major cause of bovine mastitis and respiratory disease, leading to substantial economic losses and increased antimicrobial use in the dairy industry. Because conventional culture and susceptibility testing are time-consuming, empirical treatment is common, often resulting in therapeutic failure and the emergence of multidrug-resistant strains. This study developed a rapid diagnostic platform using multiplex PCR and nanopore sequencing to predict antimicrobial susceptibility within only 6 h. Our analysis of 329 Japanese isolates reveals a significant temporal increase in mutations associated with resistance to macrolides, tetracyclines, and fluoroquinolones between 2013–2016 and 2023–2025. This cost-effective method enables simultaneous analysis of multiple samples, providing a practical tool for evidence-based clinical decision-making. These findings highlight the urgent need for molecular surveillance to monitor and control the spread of highly resistant *M. bovis* lineages in global cattle populations.

## INTRODUCTION

*Mycoplasma bovis* is a global pathogen that causes respiratory diseases, mastitis, and arthritis, leading to substantial economic losses (1). As a primary component of the bovine respiratory disease complex, it particularly affects calves, causing chronic pneumonia, which results in reduced growth rates and high mortality. In Japan, *M. bovis* is the leading cause of mycoplasma mastitis in dairy cattle. This condition is characterized by severe inflammation, high transmissibility, and poor response to treatment, placing a significant economic burden on the dairy industry (2).

Given the lack of effective vaccines, disease control primarily relies on antimicrobial therapy (3). However, *Mycoplasma* species lack cell walls and are therefore intrinsically resistant to β-lactam antimicrobials, leaving macrolides, lincosamides, tetracyclines, phenicols, and fluoroquinolones as the main therapeutic options (4). Although the treatment of bacterial infections ideally depends on the isolation of the causative bacteria and antimicrobial susceptibility testing, *Mycoplasma* species grow slowly, requiring approximately 1 week for culture and an additional week for susceptibility testing (4). Consequently, empirical therapy is commonly practiced in clinical settings, making it difficult to select effective antimicrobials and often leading to inappropriate antimicrobial use. This treatment paradigm contributes to ongoing economic losses and the emergence of antimicrobial-resistant strains. Therefore, the development of rapid methods for pathogen identification and antimicrobial susceptibility testing is essential.

Resistance-associated mutations in *rrl* and *rrs* confer resistance to tetracyclines, macrolides, lincosamides, and amphenicols, whereas specific mutations in *gyrA* and *parC* result in reduced susceptibility to fluoroquinolones in *M. bovis* (5–7). The detection of these mutations offers a promising strategy for the rapid prediction of antimicrobial susceptibility. Hybridization probe-based susceptibility prediction methods combined with Sanger sequencing have been reported for *M. bovis* (5). However, these approaches require the integration of multiple analytical procedures, making them technically complex and cumbersome.

We previously developed a rapid diagnostic platform using nanopore sequencing for the identification of pathogens causing mastitis and respiratory diseases, enabling bacterial species identification within approximately 6 h from sampling (8, 9). Based on this, we aimed to establish a nanopore-based genetic method for the rapid prediction of antimicrobial susceptibility in *M. bovis*.

In this study, we analyzed *M. bovis* isolates collected in Japan to characterize resistance-associated mutations using multiplex PCR-based nanopore sequencing and to infer antimicrobial susceptibility profiles. We further assessed temporal trends between isolates collected during 2013–2016 and 2023–2025, providing insight into recent changes and establishing a framework for rapid, genetics-based antimicrobial guidance in clinical practice.

## MATERIALS AND METHODS

### Bacterial strains

A total of 329 *M. bovis* isolates were used in this study. These isolates were collected as part of routine diagnostic submissions to a veterinary diagnostic laboratory (NDTS Co., Ltd.). The isolates were obtained from nasal swabs (*n* = 267), mastitis milk (*n* = 45), synovial fluid (*n* = 5), and other sources (*n* = 12), collected between 2013 and 2016 (*n* = 112) and between 2023 and 2025 (*n* = 217). Samples were collected from 60 farms across multiple regions (19 prefectures) in Japan.

The isolation and identification procedures are described below. Nasal swab samples were directly inoculated into 2 mL of modified PPLO (NK) broth (Kanto Chemical Co., Inc., Tokyo, Japan) and incubated at 37°C for 72 h. The NK broth is a modified Hayflick-type medium based on PPLO broth supplemented with horse serum, fresh yeast extract, DNA, and selective agents (penicillin G and thallium acetate). Liquid samples, including milk and synovial fluid, were inoculated at a volume of 100 μL into the same broth and incubated under identical conditions. Subsequently, the broth culture was streaked onto modified PPLO (NK) agar plates (Kanto Chemical Co., Inc.) and incubated at 37°C for 5–10 days in a 5% CO₂ incubator. Colony formation was examined, and a single colony was selected and inoculated into the *Mycoplasma* (NK) broth medium. Following incubation at 37°C for 72 h, the presence of *M. bovis* was confirmed by PCR using a commercial detection kit (Kanto Chemical Co., Inc.; Cica Geneus *Mycoplasma bovis* Detection Plus kit) according to the manufacturer’s instructions. The kit contains proprietary primers and reagents, and the primer sequences are not disclosed by the manufacturer.

### Determination of minimum inhibitory concentrations (MICs)

The antimicrobial susceptibilities of *M. bovis* isolates were evaluated against seven antimicrobials: one 16-membered macrolide (tylosin), two 15-membered macrolides (gamithromycin and tulathromycin), lincomycin, florfenicol, oxytetracycline, and enrofloxacin. For quality control and comparative analysis, *M. bovis* PG45ᵀ was included in all assays. Antimicrobials were purchased from the following suppliers: tylosin (Fujifilm Wako Pure Chemical, Tokyo, Japan), gamithromycin (Selleck Chemicals, Houston, TX), tulathromycin A (Selleck Chemicals), lincomycin (Sigma-Aldrich, St. Louis, MO), florfenicol (Tokyo Chemical Industry Co., Ltd., Tokyo, Japan), oxytetracycline (Tokyo Chemical Industry Co., Ltd.), and enrofloxacin (Sigma-Aldrich). Each antimicrobial was dissolved according to its physicochemical properties. Water-soluble compounds (tylosin, lincomycin, oxytetracycline, and enrofloxacin) were dissolved in sterile distilled water. Poorly water-soluble compounds (gamithromycin, tulathromycin A, and florfenicol) were dissolved in dimethyl sulfoxide. All stock solutions were filter-sterilized through 0.22 µm membranes and stored at −80°C until use.

MICs for all isolates were determined using the broth microdilution method with a modified protocol based on the guidelines of the Clinical and Laboratory Standards Institute (10), with methodological reference to the Japanese Society of Antimicrobials for Animals for antimicrobial susceptibility testing of animal-derived *Mycoplasma* and *Ureaplasma* (11). Briefly, MIC testing plates were prepared in 96-well microplates by dispensing 100 μL of antimicrobial-containing medium into each well to obtain final antimicrobial concentrations of 0, 0.125, 0.25, 0.5, 1, 2, 4, 8, 16, 32, 64, and 128 μg/mL. A fully grown culture showing a color change from red to yellow in phenol red-containing medium was used for inoculum preparation. Briefly, 100 μL of the culture was added to 3 mL of NK broth medium (Kanto Chemical Co., Inc.) to prepare the inoculum suspension. Because accurate colony counting of mycoplasmas is technically difficult, the inoculum size was adjusted to approximately 10⁴–10⁵ color-changing units/mL by dilution of the fully grown culture. Subsequently, 10 μL of the inoculum suspension was added to each well, and the plates were incubated at 37°C for 3 days. Following incubation, color changes in each well were visually examined, and the MIC was defined as the lowest antimicrobial concentration showing no color change.

### Detection of point mutations associated with antimicrobial susceptibility using multiplex PCR-based nanopore sequencing

Point mutations associated with antimicrobial susceptibility were detected by nanopore sequencing targeting the *gyrA, parC*, and *rrs-rrl* regions of *M. bovis*. Primers were designed based on the genome sequence of *M. bovis* PG45^T^ (accession no. CP002188), and the primer sequences and expected amplicon sizes are listed in Table S1. The expected amplicon lengths were approximately 400 bp for *gyrA*, 530 bp for *parC*, and 3,364 bp for the *rrs-rrl* region. *M. bovis* possesses two *rrn* operons (*rrnA* and *rrnB*), and the primers targeting the *rrs-rrl* region were designed based on conserved sequences identical in both operons of the reference strain PG45^T^. Therefore, the resulting amplicons likely represented a mixture of both operons.

Multiplex PCR was performed using MightyAmp DNA Polymerase (TaKaRa Bio Inc., Shiga, Japan), which was selected because of its suitability for direct PCR and high tolerance to inhibitors, enabling efficient amplification of long targets, including the *rrs-rrl* region. Cultured *M. bovis* isolates were used directly as PCR templates without prior DNA extraction. The reaction mixture (25 μL total volume) consisted of 12.5 μL of 2× MightyAmp Buffer, 0.5 μL of MightyAmp polymerase, 10 μL of distilled water, 0.075 μL each of 100 μM forward and reverse primers for *gyrA* and *parC*, 0.075 μL each of 100 μM primers targeting the *rrs-rrl* region (final concentration of all primers, 0.3 μM), and 1 μL of template culture.

Thermal cycling conditions were as follows: initial denaturation at 98°C for 2 min; 35 cycles of denaturation at 98°C for 10 s, annealing at 52°C for 15 s, and extension at 68°C for 3 min; followed by a final extension at 68°C for 5 min. PCR products were confirmed by electrophoresis on 1.0% agarose gels alongside a DNA size marker and visualized under UV illumination after staining with a nucleic acid dye.

To ensure reliable amplification, isolates that failed to yield sufficient PCR products on the first attempt were retested using a 1:10 dilution of the culture broth as the PCR template. Using this approach, target amplicons were successfully obtained from more than 95% of isolates on the first attempt, and the remaining isolates were successfully amplified after dilution.

PCR amplicons were purified using AMPure XP beads (Beckman Coulter). Briefly, an equal volume of AMPure XP beads was added to the PCR product and mixed thoroughly. After incubation at room temperature for 5 min, tubes were placed on a magnetic stand for 2 min, and the supernatant was discarded. Beads were washed twice with 70% ethanol, air-dried, and DNA was eluted in 10 μL of distilled water. DNA concentration was measured using a Qubit 4 Fluorometer (Thermo Fisher Scientific, Waltham, MA), and samples were adjusted to 40 ng/μL when possible.

Barcoding and library preparation were performed using a Rapid Barcoding Sequencing Kit (SQK-RBK114.24; Oxford Nanopore Technologies) according to the manufacturer’s instructions. For each sample, 3.5 μL of purified amplicon was mixed with 0.5 μL of rapid barcode and incubated at 30°C for 2 min, followed by 80°C for 2 min, and immediately cooled on ice. Up to 24 barcoded samples were pooled and purified again using AMPure XP beads at a 1:1 ratio, followed by two washes with 75% ethanol. DNA was eluted in 10 μL of elution buffer, and 5.5 μL was used for adapter ligation. Rapid Adapter was diluted by mixing 0.15 μL of adapter with 0.35 μL of adapter dilution buffer, and 0.5 μL of the diluted adapter was added to the pooled library and incubated at room temperature for 5 min.

Flongle flow cells R10.4.1 (FLO-FLG114; Oxford Nanopore Technologies) were primed with 120 μL of priming mixture consisting of 117 μL of Flow Cell Flush and 3 μL of Flow Cell Tether according to the manufacturer’s instructions. A sequencing mixture (30 μL total volume) consisting of 15 μL of sequencing buffer, 10 μL of loading beads, and 5 μL of the final library was loaded onto the primed flow cell. Sequencing was performed using the default run settings and continued until the number of output reads reached a plateau (approximately 3 h).

Sequence data were basecalled and demultiplexed by barcode. FASTQ files were imported into Geneious software (Tomy Digital Biology Co., Ltd.), and reads were mapped to the reference sequences of the *gyrA, parC*, and *rrs-rrl* regions of *M. bovis* PG45^T^. Targeted amplicon sequencing yielded a high read depth, with the majority of regions achieving a coverage of over 1,000×. Antimicrobial susceptibility-associated point mutations were identified by comparison with the reference sequences, and consensus sequences were generated using a strict minimum sequencing coverage of 10× to ensure variant call reliability, even in samples with lower amplification efficiency. Because the amplified *rrs-rrl* products likely represented a mixture of both *rrn* operons, mutations present in only one operon could also be detected when supported by sufficient sequencing reads, although operon-specific analysis was not performed in this study. An example of mutation detection using Geneious software is shown in Fig. S1.

### Statistical analysis

Regarding the association between antimicrobial susceptibility and resistance-associated genetic mutations, statistical analyses were performed for mutation groups with sufficient sample sizes. However, because several groups were highly unbalanced or contained very small numbers of isolates, some comparisons were analyzed descriptively only. Statistical analyses of MIC distributions among mutation groups with sufficient sample sizes were performed using the Kruskal-Wallis test followed by Mann-Whitney *U*-tests for pairwise comparisons. Differences in the distribution of antimicrobial susceptibility categories during 2013–2016 and 2023–2025 were assessed using the χ^2^ test of independence in GraphPad Prism version 8 (MDF Co., Ltd., Tokyo, Japan).

## RESULTS

### Antimicrobial susceptibility of the tested *M. bovis* strains

Among the preserved *M. bovis* isolates, some exhibited poor growth after storage. Consequently, antimicrobial susceptibility testing was successfully performed for only 277 of the 329 isolates.

The MIC distributions for the tested antimicrobials among the 277 isolates are summarized in Table 1. For the macrolides, tylosin, gamithromycin, and tulathromycin, the MIC_50_ values were 64, 16, and 1 mg/L, respectively, whereas the MIC_90_ values for all three antimicrobials exceeded 128 mg/L. For lincomycin, the MIC_50_ was 4 mg/L, and the MIC_90_ was >128 mg/L. Florfenicol showed an MIC_50_ of 8 mg/L and an MIC_90_ of 16 mg/L. The MIC_50_ and MIC_90_ values of oxytetracycline were 32 and 64 mg/L, respectively, and those of enrofloxacin were 16 and 64 mg/L, respectively.

**TABLE 1:** Distribution of MICs for 277 *M. bovis* isolates with available antimicrobial susceptibility results and MIC of *M. bovis* PG45^*a*^

	MIC (mg/L)												MIC_50_ (mg/L)	MIC_90_ (mg/L)	Range (mg/L)	MIC of PG45^T^ (mg/L)
Antimicrobials	≤0.125	0.25	0.5	1	2	4	8	16	32	64	128	>128				
Tylosin						1	7	16	47	82	38	86	64	>128	4–>128	0.5
Gamithromycin			2	2	15	31	71	38	20	27	21	50	16	>128	0.5–>128	4
Tulathromycin	35	52	34	45	13	4	5	4	12	16	25	32	1	>128	≤0.125–>128	0.25
Lincomycin			11	42	83	33	19	7	1	8	40	33	4	>128	0.5–>128	1
Florfenicol					8	65	120	67	5	10	1	1	8	16	2–>128	4
Oxytetracycline				1		8	47	69	65	84	3		32	64	1–128	1
Enrofloxacin	1	8	20	12	26	19	21	48	54	65	3		16	64	≤0.125–128	0.5

^*a*^ MICs, minimum inhibitory concentrations.

Because official breakpoints have not been established for *M. bovis*, and EUCAST epidemiological cutoff values (ECOFFs) are still unavailable for most of the antimicrobials tested, a formal overall resistance rate could not be determined in this study (12).

The MIC values obtained for *M. bovis* PG45^T^ were generally comparable to those reported previously, although differences were observed for several antimicrobial agents. Such discrepancies may reflect differences in MIC determination methods among studies, including variations in culture media, inoculum size, incubation conditions, and endpoint interpretation criteria (13, 14). Similar inter-study variability in MIC values for *M. bovis* reference strains has been reported previously (13, 15).

### Association between antimicrobial susceptibility and resistance-associated genetic mutations

Nanopore sequencing of multiplex PCR amplicons enabled the detection of sequence differences relative to the reference strain *M. bovis* PG45^T^ across the entire *rrl-rrs* region, as well as *gyrA* and *parC*. Using this approach, mutations in the target regions were identified in 329 *M. bovis* isolates.

**Fig 1:**
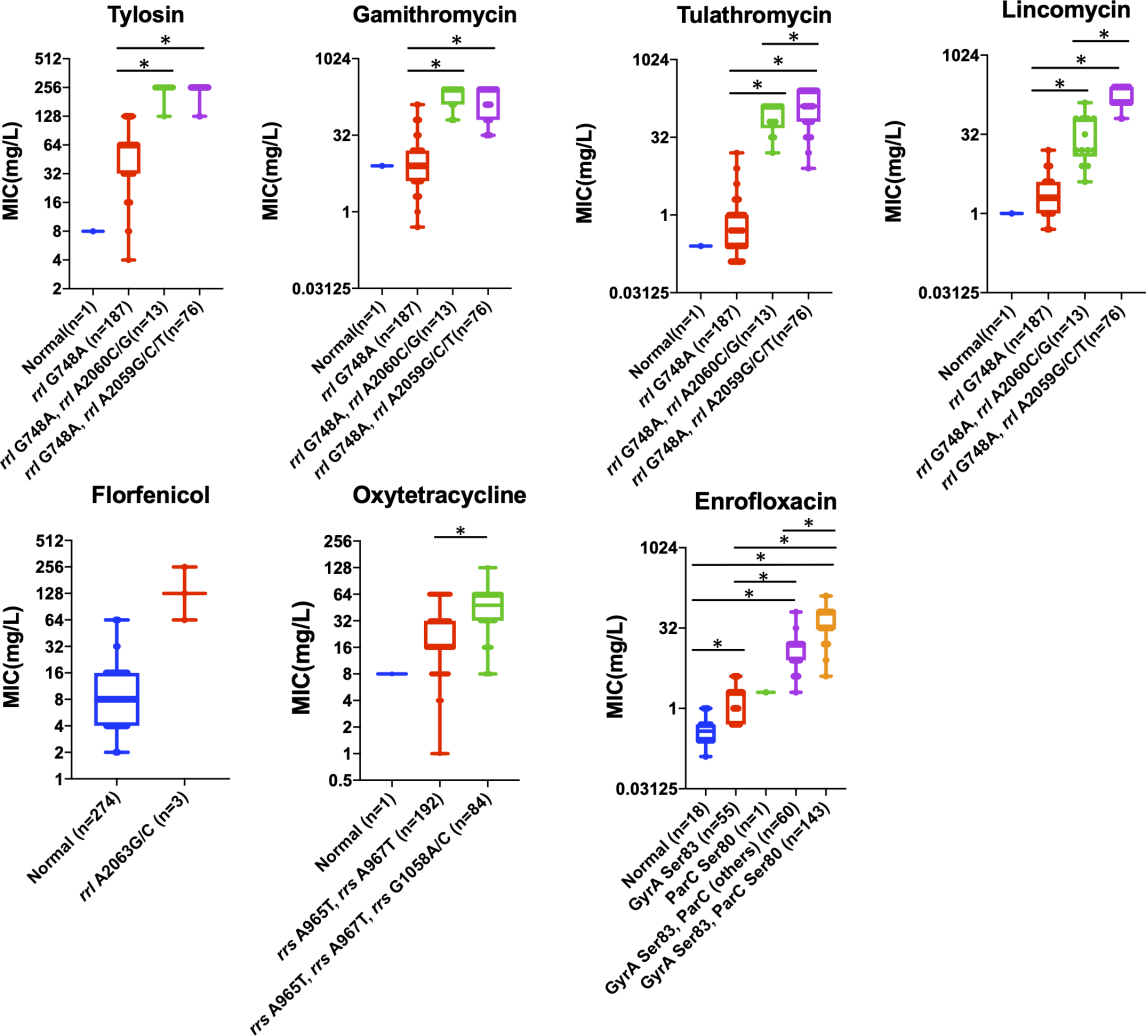
Association between mutations in target genes and antimicrobial MICs. Box-and-whisker plots illustrate the distribution of the MIC values for each antimicrobial agent. The boxes represent the median and interquartile range, and the whiskers extend to the maximum and minimum values. Each dot represents an individual isolate, and the horizontal lines indicate the median. Statistical analyses were performed for mutation groups with sufficient sample sizes using the Kruskal-Wallis test, followed by Mann-Whitney *U*-tests for pairwise comparisons. Groups with very small sample sizes were analyzed descriptively only. *P < 0.05.

For the 277 isolates for which antimicrobial susceptibility data were available, the relationships between the MIC values for each antimicrobial agent and the corresponding genetic mutations were visualized (Fig. 1). For plotting purposes, MIC values ≤0.125 mg/L were assigned a value of 0.125 mg/L, and MIC values >128 mg/L were assigned a value of 256 mg/L.

Mutations at positions G748, A2059, and A2060 in *rrl* are known to be associated with susceptibility to macrolides and lincosamides (5, 6). In this study, all but one isolate harbored the G748 mutation, precluding the assessment of its independent effect on susceptibility to these antimicrobials. Isolates carrying only the G748 mutation (*n* = 187) exhibited a wide range of MIC values for macrolides and lincosamides (Fig. 1). Therefore, the presence of the G748A mutation alone could not be conclusively interpreted as indicating complete non-susceptibility in this study. In contrast, isolates with an additional A2060 (*n* = 13) or A2059 (*n* = 76) mutation in combination with G748 showed higher MIC values for macrolides and lincomycin than isolates carrying the G748 mutation alone (Fig. 1). Statistical analyses demonstrated that isolates carrying additional A2060 or A2059 mutations exhibited significantly higher MIC values for all tested macrolides and lincomycin compared with isolates carrying the G748 mutation alone (*P* < 0.05). For tulathromycin and lincomycin, isolates carrying the A2059 mutation showed significantly higher MIC values than those carrying the A2060 mutation (*P* < 0.05).

The A2063 mutation in *rrl* has been known to influence susceptibility to amphenicol antimicrobials (6). Although this mutation was detected in only three isolates in this study, these isolates showed higher MIC values for florfenicol than the other isolates (Fig. 1). Because of the limited number of isolates carrying this mutation, statistical analysis was not performed.

Mutations at positions A965, A967, and G1058 in *rrs* are known to be associated with susceptibility to tetracyclines (5, 6). In this study, all but one isolate carried A965 and A967 mutations, making it impossible to evaluate the independent contribution of these mutations to tetracycline susceptibility. Therefore, the effect of A965 and A967 alone on tetracycline susceptibility could not be determined in this study. However, isolates carrying an additional G1058 mutation in combination with A965 and A967 (*n* = 84) showed significantly higher MIC values for oxytetracycline than isolates lacking the G1058 mutation (*n* = 192) (*P* < 0.05).

Amino acid-altering mutations in *gyrA* and *parC* are known to be associated with susceptibility to fluoroquinolones (5, 6). Isolates with a single mutation in GyrA (Ser83) (*n* = 55) showed significantly higher MIC values for enrofloxacin than isolates without these mutations (*P* < 0.05). One isolate carried a single ParC (Ser80) mutation; therefore, statistical analysis was not performed for this group. Significantly higher MIC values were observed in isolates carrying dual mutations in *gyrA* and *parC* compared with isolates carrying a single GyrA mutation (*P* < 0.05). Among the isolates with dual mutations, ParC substitutions were detected at Ser80, Thr81, and Asp84. Isolates carrying dual mutations including ParC (Ser80) (*n* = 143) exhibited significantly higher MIC values for enrofloxacin than isolates carrying dual mutations with ParC substitutions other than Ser80 (*n* = 60) (*P* < 0.05).

Based on available EUCAST ECOFFs (12) for florfenicol and enrofloxacin, isolates harboring resistance-associated mutations were predominantly classified as non-wild-type populations (Table S2).

### Prediction of antimicrobial susceptibility based on genetic mutations

Based on the above results, antimicrobial susceptibility was predicted according to the genetic mutations in all tested isolates (*n* = 329).

Regarding *rrl* mutations associated with susceptibility to macrolides and lincomycin, only one isolate (0.3%) shared an identical sequence with the reference strain *M. bovis* PG45^T^.

A total of 232 isolates (70.5%) carried the G748 mutation alone, 13 (4.0%) carried both G748 and A2060 mutations, and 83 (25.2%) carried both G748 and A2059 mutations. Overall, isolates predicted to have reduced susceptibility to macrolides and lincomycin due to additional A2060 or A2059 mutations accounted for 29.2% of all isolates.

The *rrl* A2063 mutation associated with florfenicol susceptibility was detected in three of the 329 isolates (0.9%).

Regarding *rrs* associated with oxytetracycline susceptibility, only one isolate (0.3%) showed an identical sequence to *M. bovis* PG45^T^, whereas 237 isolates (72.0%) harbored A965 and A967 mutations. In addition, 91 isolates (27.7%) carried the G1058, A965, and A967 mutations and were predicted to have reduced susceptibility to tetracyclines.

With respect to *gyrA* and *parC* mutations associated with fluoroquinolone susceptibility, 22 isolates (6.7%) had amino acid sequences identical to those of *M. bovis* PG45^T^. Sixty-six isolates (20.0%) carried a single GyrA Ser83 mutation, and one isolate (0.3%) carried a single ParC Ser80 mutation, both associated with reduced susceptibility to enrofloxacin. Dual mutations associated with particularly low susceptibility to enrofloxacin were observed in 73 isolates (22.2%) carrying GyrA Ser83 and ParC mutations other than Ser80 and in 167 isolates (50.8%) carrying GyrA Ser83 plus ParC Ser80 mutations. Overall, 93.3% of the isolates harbored at least one mutation affecting antimicrobial susceptibility, whereas dual mutations associated with marked reductions in susceptibility were detected in 72.9% of the isolates.

### Trends in antimicrobial susceptibility based on resistance-associated mutations (2013–2016 vs 2023–2025)

The overall proportion of mutations associated with antimicrobial susceptibility was compared between isolates collected in 2013–2016 (*n* = 112) and those collected in 2023–2025 (*n* = 217).

**Fig 2:**
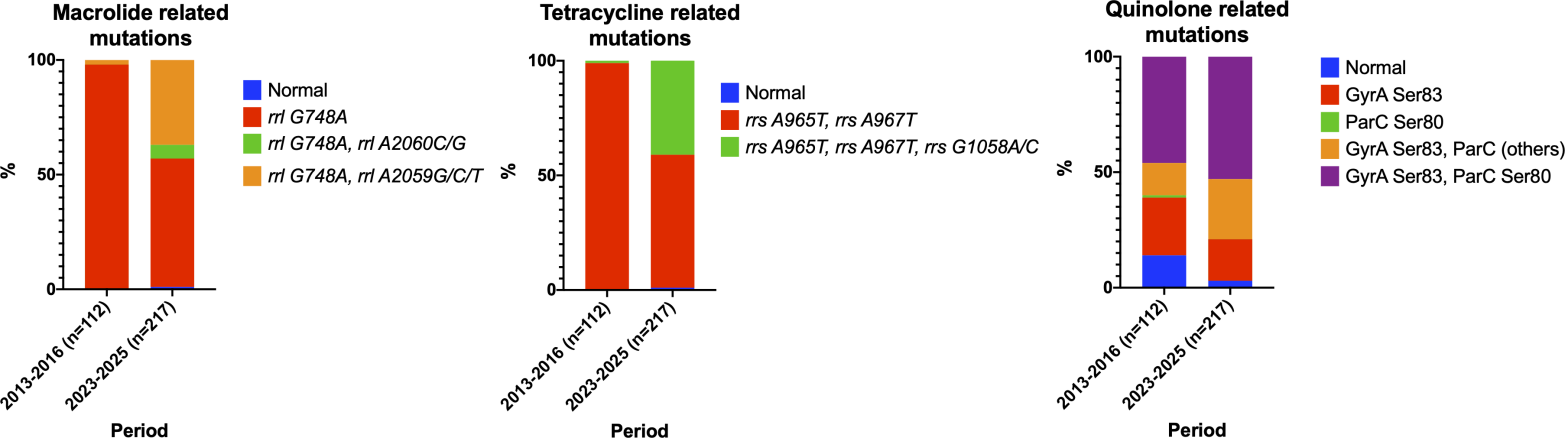
Proportions of point mutations associated with antimicrobial susceptibility to macrolides, tetracyclines, and quinolones in *Mycoplasma bovis* isolates (*n* = 329) collected during 2013–2016 and 2023–2025.

Regarding mutations in *rrl* associated with susceptibility to macrolides and lincomycin, only two isolates (1.8%) obtained during 2013–2016 carried additional A2060 or A2059 mutations together with G748, whereas 94 of 217 isolates (43.3%) obtained during 2023–2025 harbored these mutations (Fig. 2). These mutations showed a significant increase from 2013–2016 to 2023–2025 (*P* < 0.001).

The *rrl* A2063 mutation, associated with florfenicol susceptibility, was detected in three isolates, all of which were obtained in 2023 or 2024.

Regarding *rrs* mutations associated with oxytetracycline susceptibility, only one isolate (0.9%) obtained during 2013–2016 carried the G1058 mutation, in addition to A965 and A967, whereas 90 of the 217 isolates (41.5%) obtained during 2023–2025 harbored this mutation (Fig. 2). These mutations increased significantly between the two periods (*P* < 0.001).

For fluoroquinolone-associated mutations in *gyrA* and *parC*, 14.3% of isolates collected during 2013–2016 shared identical amino acid sequences with *M. bovis* PG45^T^, compared with only 2.7% of isolates collected during 2023–2025, indicating that 97.3% of the recent isolates carried at least one susceptibility-associated mutation (Fig. 2). Isolates harboring dual mutations associated with marked reductions in susceptibility increased from 59.8% in 2013–2016 to 79.8% in 2023–2025. These mutations have shown a significant increase over time (*P* < 0.001).

The trends stratified by origin are shown in Fig. S2 (267 nasal swab and 45 mastitis milk isolates). Although the number of mastitis milk isolates was limited, they exhibited resistance-associated mutation trends similar to those observed in the nasal swab isolates.

Comparison of MIC_50_ and MIC_90_ values between isolates collected during 2013–2016 and 2023–2025 demonstrated clear upward shifts in MIC distributions for several antimicrobials, particularly gamithromycin, tulathromycin, lincomycin, and enrofloxacin (Table S3).

## DISCUSSION

This study developed a rapid genotype-based method to estimate the antimicrobial susceptibility-associated profile of *M. bovis* by combining multiplex PCR with nanopore sequencing, enabling the simultaneous detection of mutations in *rrs*, *rrl*, *gyrA*, and *parC*. This approach allowed the rapid prediction of susceptibility to multiple antimicrobial classes, including macrolides, lincosamides, amphenicols, tetracyclines, and fluoroquinolones, within approximately 6 h from the start of testing. Although several molecular methods for predicting antimicrobial susceptibility in *M. bovis* have been reported previously (5, 16), our approach is simpler and enables the rapid detection of a broader range of antimicrobials and mutation sites. Even previously unreported mutations in antimicrobial target genes can be detected using this approach; however, the clinical significance of newly identified mutations would require further phenotypic and functional validation. Furthermore, by analyzing 24 samples simultaneously on a compact and relatively low-cost Flongle flow cell, this method achieves substantial cost reduction in per-sample sequencing cost. A single Flongle flow cell currently costs approximately US$90–120, resulting in a sequencing cost of only a few US dollars per sample when 24 samples are multiplexed in a single run.

Analysis of the relationship between antimicrobial susceptibility and genetic mutations showed that isolates exhibiting mutations at *rrl* G748, together with additional substitutions at A2060 or A2059, exhibited low susceptibility to macrolides and lincomycin. The A2060 mutation had a weaker impact on susceptibility than did the A2059 mutation. High-level resistance to macrolides and lincomycin requires additional mutations at A2060 or A2059 in combination with G748 (5, 6). Based on a limited number of isolates, strains harboring the *rrl* A2063 mutation displayed markedly reduced susceptibility to florfenicol. Although the involvement of *rrl* mutations in florfenicol resistance has been suggested previously (6), this study identified A2063 mutant field isolates with low florfenicol susceptibility. Isolates carrying mutations at *rrs* A965 and A967 showed reduced susceptibility to tetracyclines, which was further decreased in strains harboring the G1058 mutation. These three mutations have been previously associated with tetracycline susceptibility (7).

Enrofloxacin susceptibility correlated with amino acid substitutions in GyrA and ParC. Mutations within the quinolone resistance-determining regions of these genes have been shown to reduce quinolone susceptibility, with double mutations conferring even lower susceptibility (5), whereas mutations in *gyrB* and *parE* have little impact (17). These findings demonstrate that the antimicrobial susceptibility of *M. bovis* can be reliably inferred by detecting specific genetic mutations.

We further compared the trends in resistance-associated mutations between isolates collected in 2013–2016 and 2023–2025. Mutations associated with macrolide resistance (*rrl* G748) and tetracycline resistance (*rrs* A965 and A967) were present in nearly all isolates during 2013–2016. These mutations are characteristic genetic features of the globally disseminated ST5 lineage, including in Japan (5, 7), indicating that such strains had already spread widely in Japan by that period. This suggests that, as early as 2013, tetracyclines and 16-membered macrolides were likely to have reduced clinical efficacy, which is consistent with previous reports indicating that these antimicrobials were no longer suitable as first-line treatments for *M. bovis* infections (6, 14, 18).

Isolates collected during 2023–2025 showed a significant reduction in susceptibility to macrolides, tetracyclines, and fluoroquinolones compared to those collected from 2013 to 2016. Frequent administration of tetracyclines, macrolides, and fluoroquinolones likely contributed to this shift, as the spread of antimicrobial resistance is closely linked to antimicrobial use. Moreover, the introduction of 15-membered macrolides, such as tulathromycin, after 2013 may have promoted the dissemination of highly macrolide-resistant strains harboring *rrl* A2060 or A2059 mutations. Consistently, *M. bovis* isolates from Canada exhibited tulathromycin resistance rates exceeding 90%, suggesting an association with its clinical use (19). Additionally, florfenicol-low-susceptibility isolates were detected among the strains collected in 2023 and 2024, highlighting the need for continued vigilance regarding the future emergence and spread of florfenicol resistance.

As most mycoplasmas lack plasmids, antimicrobial pressure is presumed to drive the clonal expansion of specific resistant lineages and the dissemination of particular *M. bovis* types (5, 20). Consequently, multidrug-resistant strains are now widespread in the Japanese cattle population, highlighting the urgent need for measures to control their spread.

The susceptibility trends observed in this study are broadly consistent with, but in some respects more severe than, those reported previously in Japan and internationally. In Japan, the near-universal presence of *rrl* G748A and *rrs* A965T/A967T mutations among our 2013–2016 isolates corroborates earlier findings that ST5-group strains—dominant in Japan since 1999—were already characterized by low susceptibility to 16-membered macrolides and tetracyclines (5). Regarding fluoroquinolones, an earlier study from Hokkaido showed that dual GyrA-ParC mutations were present in only a minority of isolates (21), whereas in the present study such dual mutations rose to 79.8% by 2023–2025, suggesting markedly increased selective pressure from fluoroquinolone use. Internationally, the picture varies by region and antimicrobial use practice. Canadian feedlot isolates showed tulathromycin resistance exceeding 90% (19), paralleling the high-level macrolide resistance now seen in Japan, while Australian isolates retained low MICs for fluoroquinolones and most macrolides—likely reflecting Australia’s prohibition on fluoroquinolone use in food-producing animals (22). In Europe, French isolates showed progressive multi-class susceptibility decline over 30 years (14), and Spanish isolates were broadly resistant to macrolides, lincosamides, and tetracyclines, with fluoroquinolone susceptibility varying by sequence type (23, 24). As recently reviewed by Sulyok et al. (16), notable variability in MIC data across regions underscores the importance of region-specific surveillance—a conclusion strongly supported by the steep, Japan-specific temporal increases in multidrug resistance documented here.

This study has several limitations. First, the susceptibility prediction model based on genetic mutations was developed using isolates collected from multiple regions in Japan; however, the data set was limited to samples submitted to a single diagnostic laboratory, and its applicability to other geographical or clinical settings therefore remains to be confirmed. In addition, this study primarily analyzed cultured isolates, and the performance of the method for direct detection from clinical specimens has not yet been evaluated. Consequently, additional validation is warranted to assess its sensitivity and specificity in the presence of mixed microbial populations or host-derived DNA, as well as its robustness and practicality for routine diagnostic implementation, including considerations of cost and workflow. Moreover, although strong associations were observed between several resistance-associated mutations and elevated MIC values, genotype-based prediction does not fully substitute for conventional phenotypic susceptibility testing, particularly for isolates carrying uncommon mutation patterns or resistance mechanisms not targeted in this study. Therefore, the present approach should be interpreted as a rapid supportive tool for estimating reduced antimicrobial susceptibility rather than a definitive standalone method for clinical susceptibility determination.

Furthermore, although nanopore sequencing accuracy has improved substantially in recent years, its per-base accuracy remains lower than that of short-read sequencing platforms. This limitation may particularly affect the detection of point mutations in low-complexity genomic regions, including the AT-rich regions characteristic of *Mycoplasma* genomes. In this study, to mitigate the impact of sequencing errors, we performed targeted amplicon sequencing to achieve high read depth, with most target regions yielding a coverage of over 1,000×. Consensus sequences were generated using strict minimum coverage of 10× even for low-amplification samples. While the strong correlation between the detected mutations and phenotypic MIC values supports the reliability of our data, the potential influence of nanopore-specific sequencing errors in low-coverage or highly repetitive regions should still be considered when interpreting mutation data.

In addition, although this method enables the detection of sequence variations in antimicrobial target genes, interpretation of resistance phenotypes currently depends on previously characterized genotype-phenotype associations. *Mycoplasma* species exhibit relatively high mutation rates, and newly identified or previously unreported mutations may emerge whose contribution to antimicrobial resistance remains unclear. Therefore, additional phenotypic susceptibility testing and functional analyses may be required to determine the clinical significance of novel mutations.

In conclusion, we established a rapid and straightforward method for predicting the susceptibility of *M. bovis* to tetracyclines, macrolides, lincomycin, florfenicol, and fluoroquinolones. This nanopore-based platform enables rapid, genetics-driven antimicrobial guidance and analysis of multiple samples on a cost-effective flow cell, providing a practical tool for clinical management and surveillance of emerging multidrug-resistant *M. bovis* in dairy cattle. The application of this detection strategy is expected to facilitate the selection of effective antimicrobials and promote their appropriate use, thereby contributing to improved management of antimicrobial resistance.

## Data Availability

The data supporting the findings of this study are available within the article and its supplemental materials.

## SUPPLEMENTAL MATERIAL

Fig. S1 and S2 spectrum.01242-26-s0001.pdf Detection of the S83 mutation in gyrA using Geneious software. Multiplex PCR amplicons were mapped to the gyrA sequence of M. bovis PG45. Proportions of point mutations associated with antimicrobial susceptibility to macrolides, tetracyclines, and quinolones in M. bovis isolates collected during 2013–2016 and 2023–2025.

Table S1 spectrum.01242-26-s0002.pdf Primers used for multiplex-PCR.

Table S2 spectrum.01242-26-s0003.pdf Association between mutation groups and WT/non-WT classification based on available EUCAST ECOFF* values.

Table S3 spectrum.01242-26-s0004.pdf Comparison of MIC50 and MIC90 values for *M. bovis* isolates collected during 2013-2016 and 2023-2025.

ASM does not own the copyrights to Supplemental Material that may be linked to, or accessed through, an article. The authors have granted ASM a non-exclusive, world-wide license to publish the Supplemental Material files. Please contact the corresponding author directly for reuse.
