# Supplementary material for: Application of multiplex PCR-based nanopore sequencing for rapid detection and surveillance of antimicrobial-resistant Mycoplasma bovis in Japanese dairy cattle
Source: Microbiol Spectr. 2026 Jun 22;14(8):e01242-26. doi: 10.1128/spectrum.01242-26 (PMC13435637; doi:10.1128/spectrum.01242-26)
Supplement: Supplementary file 1 [file spectrum.01242-26-s0001.pdf]

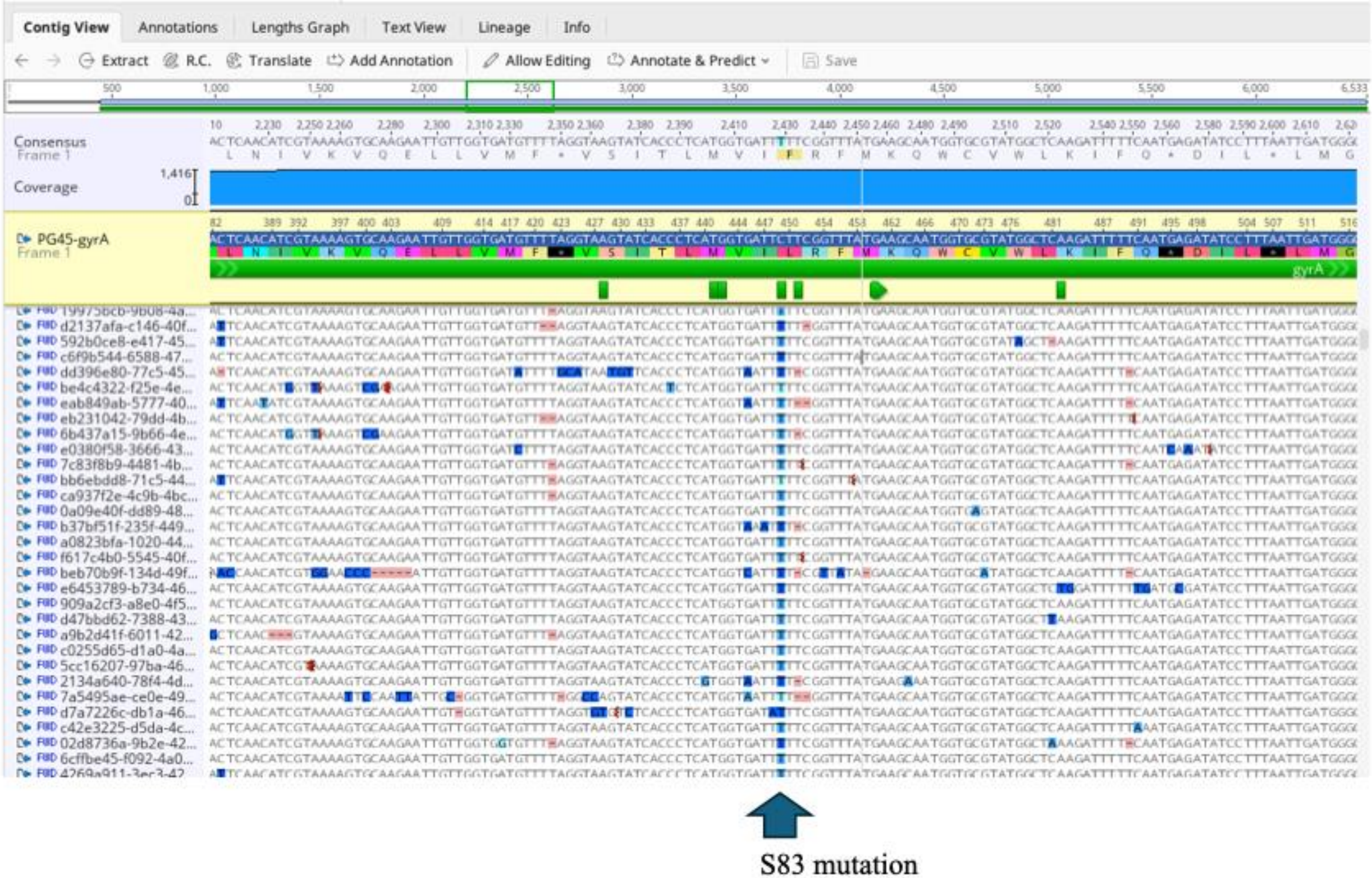


Fig. S1. Detection of the S83 mutation in *gyrA* using Geneious software. Multiplex PCR amplicons were mapped to the *gyrA* sequence of *M. bovis* PG45.

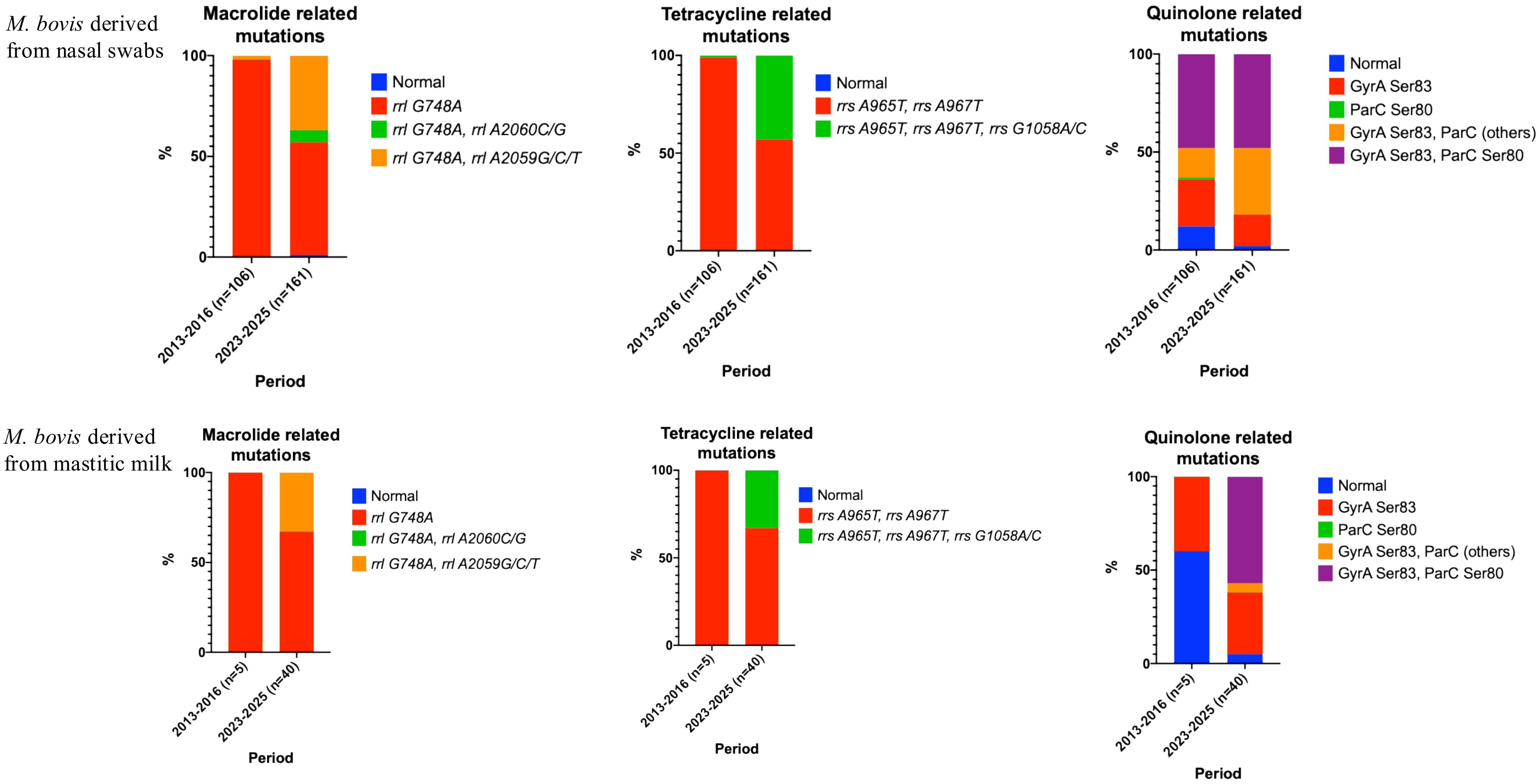


**Fig. S2. Proportions of point mutations associated with antimicrobial susceptibility to macrolides, tetracyclines, and quinolones in *M. bovis* isolates collected during 2013–2016 and 2023–2025. The upper panels show the results of 267 isolates derived from nasal swabs, and the lower panels show the results of 45 isolates derived from mastitic milk.**
