# Supplementary material for: Application of multiplex PCR-based nanopore sequencing for rapid detection and surveillance of antimicrobial-resistant Mycoplasma bovis in Japanese dairy cattle
Source: Microbiol Spectr. 2026 Jun 22;14(8):e01242-26. doi: 10.1128/spectrum.01242-26 (PMC13435637; doi:10.1128/spectrum.01242-26)
Supplement: Supplementary file 2 [file spectrum.01242-26-s0002.pdf]

Table S1. Primers used for multiplex-PCR.

	Sequence (5'-3')	Amplicon (bp)
gyrA-F	ATTGTTTCGCGTGCTCTTCC	400
gyrA-R	GAGAAGGTAACACAGAAGGCTC	
parC-F	GACGGACTTAAGCCTGTACA	530
parC-R	GGCCCTTGGATAATTTTGGC	
rrs-rrl-F	AGTCCACGCCCTAAACGATG	3364
rrs-rrl-R	TTCTGCCTTTGCGCTCTACA	
