# Supplementary material for: Application of multiplex PCR-based nanopore sequencing for rapid detection and surveillance of antimicrobial-resistant Mycoplasma bovis in Japanese dairy cattle
Source: Microbiol Spectr. 2026 Jun 22;14(8):e01242-26. doi: 10.1128/spectrum.01242-26 (PMC13435637; doi:10.1128/spectrum.01242-26)
Supplement: Supplementary file 3 [file spectrum.01242-26-s0003.pdf]

Table S2. Association between mutation groups and WT/non-WT classification based on available EUCAST ECOFF* values

Antimicrobials	Mutation group	WT n (%)	non-WT n (%)
Florfenicol	No rrl A2063 mutation	193(69.7%)	81(29.2%)
	rrl A2063 mutation	0	3 (1.1%)
Enrofloxacin	No QRDR mutation	15(5.4%)	3(1.1%)
	Single QRDR mutation	14(5.1%)	42(15.2%)
	Double QRDR mutations	0	203(73.3%)

* EUCAST ECOFF values for florfenicol and enrofloxacin were 16 and 1 mg/L, respectively.
