# Supplementary material for: Application of multiplex PCR-based nanopore sequencing for rapid detection and surveillance of antimicrobial-resistant Mycoplasma bovis in Japanese dairy cattle
Source: Microbiol Spectr. 2026 Jun 22;14(8):e01242-26. doi: 10.1128/spectrum.01242-26 (PMC13435637; doi:10.1128/spectrum.01242-26)
Supplement: Supplementary file 4 [file spectrum.01242-26-s0004.pdf]

Table S3. Comparison of MIC50 and MIC90 values for *M. bovis* isolates collected during 2013–2016 and 2023–2025.

| | 2013-2016 (n=91) | | 2023-2025 (n=186) | |
|---|---|---|---|---|
| Antimicrobials | $MIC_{50}$ (mg/L) | $MIC_{90}$ (mg/L) | $MIC_{50}$ (mg/L) | $MIC_{90}$ (mg/L) |
| Tylosin | 64 | 128 | 64 | >128 |
| Gamithromycin | 8 | 16 | 64 | >128 |
| Tulathromycin | 0.25 | 1 | 8 | >128 |
| Lincomycin | 2 | 4 | 8 | >128 |
| Florfenicol | 8 | 16 | 8 | 16 |
| Oxytetracycline | 32 | 64 | 32 | 64 |
| Enrofloxacin | 16 | 64 | 16 | 128 |
